# Reversal of blood flow in deep cerebral vein in preterm intraventricular hemorrhage: two case reports

**DOI:** 10.1186/s12887-020-02414-0

**Published:** 2020-11-11

**Authors:** Kenichi Tanaka, Rieko Sakamoto, Hiroko Imamura, Tetsuo Naramura, Shirou Matsumoto, Masanori Iwai, Hiroshi Mitsubuchi, Kimitoshi Nakamura

**Affiliations:** 1 Division of Neonatology, Kumamoto University Hospital, 1-1-1 Honjo, Chuo-ku, 860-8556 Kumamoto, Japan; 2 Department of Pediatrics, Graduate School of Life Science, Kumamoto University, 1-1-1 Honjo, Chuo-ku, 860-8556 Kumamoto, Japan

**Keywords:** Intraventricular hemorrhage, Very low birth weight infant, Internal cerebral vein, Preterm, Central venous pressure, Case report

## Abstract

**Background:**

Intraventricular hemorrhage during the early stage is a major complication in very low birth weight infants. Elevation of venous pressure is one of the contributing factors. The internal cerebral vein receives most of the venous flow from the subependymal germinal matrix, the most common site of origin of intraventricular hemorrhage. Recently, it has been reported that pulsatile or partially interrupted internal cerebral vein waveforms might also be risk factors for intraventricular hemorrhage in extremely low birth weight infants. Here, we report two cases of partially reversed internal cerebral vein flow with intraventricular hemorrhage. There are no published reports documenting this unique flow pattern.

**Case presentation:**

Between 2013 and 2020, we had in our neonatal intensive care unit two cases of very low birth weight infants (27 and 25 weeks of gestational age) who showed a partially reversed internal cerebral vein waveform pattern, which was recognized as a new blood flow pattern. Their internal cerebral vein flow patterns were continuously flat early after birth. They showed an intraventricular hemorrhage on the unilateral side with partially interrupted internal cerebral vein flow at 31 and 41 hours after birth (27- and 25-week-old neonates, respectively). Consecutively, their internal cerebral vein flow changed to a partially reversed pattern with intraventricular hemorrhage on the contralateral side at 43 and 87 hours after birth (27- and 25-week-old neonates, respectively). Their flow patterns improved by day 7. These partially reversed patterns were equivalent to triphasic venous flow, and the reverse flow corresponded to A- and V-waves.

**Conclusion:**

In the two cases, the internal cerebral vein flow patterns were normal and flat before intraventricular hemorrhage and changed to a severe flow pattern (partially interrupted or reversed flow) at the same time as the detection of intraventricular hemorrhage. After the development of intraventricular hemorrhage, they improved. These cases indicate that a partially reversed or interrupted internal cerebral vein flow pattern may be derived from central venous pressure elevation and related to intraventricular hemorrhage in very low birth weight infants, however, it is difficult to determine when this flow pattern occurs in relation to intraventricular hemorrhage.

## Background

Intraventricular hemorrhage (IVH) is a major complication in very low birth weight (VLBW) infants. However, accurate prediction and prevention of IVH have not been established yet. The germinal matrix, as a focus of IVH, is a region where abundant vascularized glial and neuronal precursor cells accumulate in the developing brain [1]. The pathogenesis of IVH is multifactorial and primarily attributed to the intrinsic vulnerability of the germinal matrix vasculature and fluctuating cerebral blood flow [1, 2]. Elevation of cerebral venous pressure is believed to be a contributing factor [2].

Recently, it has been reported that a pulsation or partial interruption of the internal cerebral vein (ICV) blood flow may be a risk factor of IVH in extremely low birth weight infants [3]. The ICV blood flow is normally a continuous flat pattern in newborns [4]. Ikeda et al. reported that the ICV blood flow is classified into four patterns: grade 0: continuous flat flow; grade 1: mild pulsatile flow (minimum speed/maximum speed ≧ 0.5); grade 2: severe pulsatile flow (minimum speed/maximum speed < 0.5); and grade 3: partially interrupted flow (minimum speed = 0 cm/s). The incidence rate of IVH was significantly higher in high-grade (2 and 3) groups than in low-grade (0 and 1) groups [3]. However, the cause of pulsation and interruption remains unclear.

The ICV composes the cerebral deep venous system and drains most of the subependymal veins into the great vein of Galen. The major source of IVH in VLBW infants is the capillary venules or small venules of the subependymal germinal matrix which are thin and fragile-walled vessels that easily cause bleeding [2, 5]. The venous blood flow from the germinal matrix drains into the subependymal veins upstream of the ICV [6]. The junction of the subependymal veins and ICV in the foramen of Monro forms a U-shaped venous angle where blood flow changes direction sharply [2]. There is a possibility that this ‘U-turn’ increases venous pressure in the germinal matrix. The anatomical peculiarity and vulnerability of the vessels may easily cause bleeding in VLBW infants.

We report two cases of IVH with a partially reversed ICV flow (PRF) as a new blood flow pattern of the ICV. This unique flow pattern has not been documented in any published reports and it could be a new predictor of IVH in VLBW infants.

## Case presentation

### Case 1

**Fig. 1:**
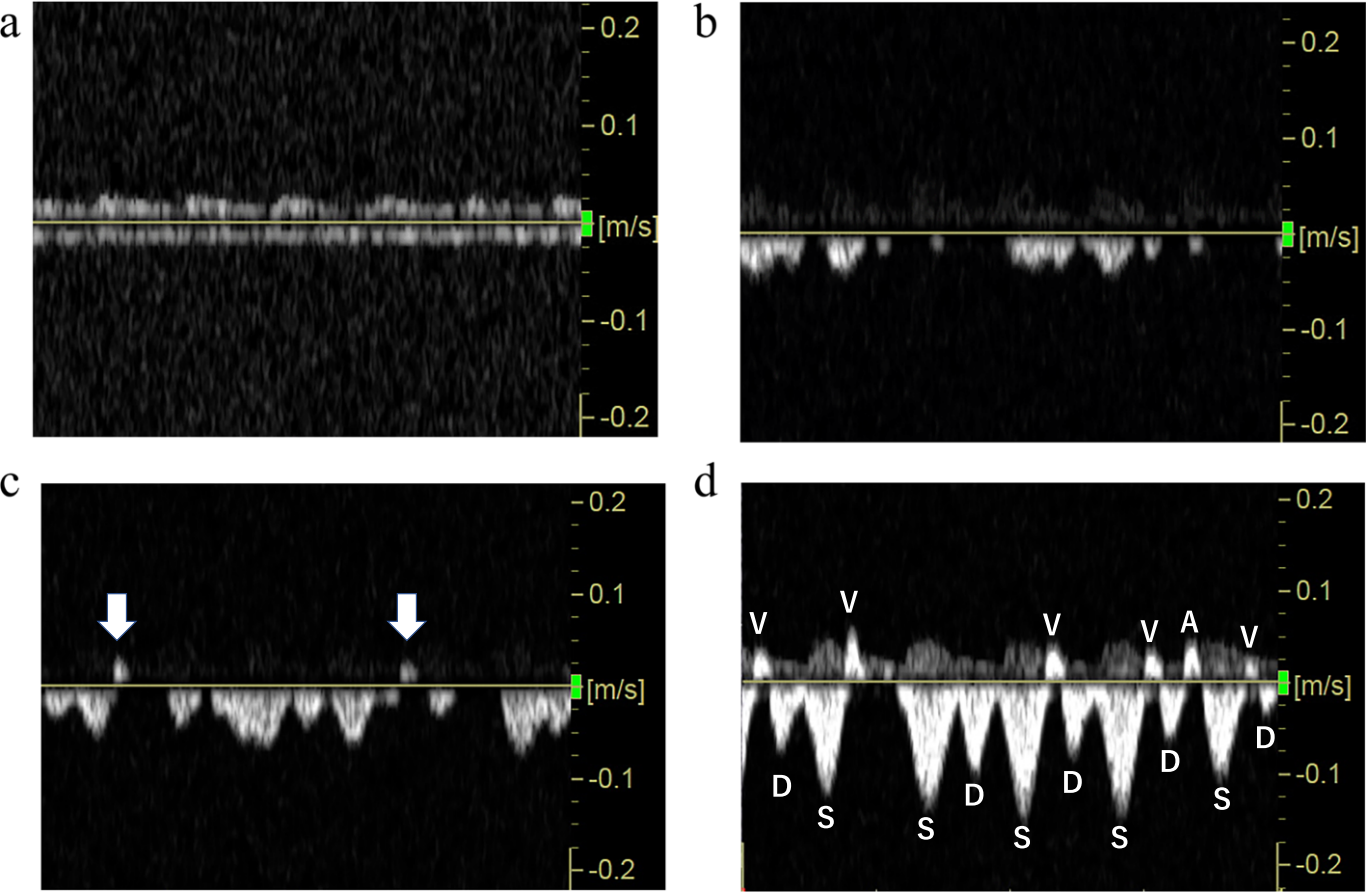
The flow of the internal cerebral vein in case 1. **a** At 10 hours after birth, the internal cerebral vein (ICV) flow pattern is continuous flat flow (grade 0). **b** Partially interrupted flow (grade 3) is confirmed at 31 hours after birth with grade III intraventricular hemorrhage (IVH) on the left side. **c** Partially reversed internal cerebral vein flow (PRF) (arrow) is recognized at 43 hours after birth with grade III bilateral IVH. **d** PRF is recognized at 90 hours after birth. The lower wave is the forward blood flow of the ICV, and the upper wave is the reverse blood flow of the ICV and the cerebral artery. The temporal relation between the perfusion flow of the cerebral artery and the ICV indicates triphasic venous pulsations constituting an A wave derived from atrial contraction, S wave derived from ventricular contraction, V wave, which corresponds to atrial overfilling, derived from ventricular contraction in the end-systolic phase, and D wave derived from ventricular dilation. The reverse flows are equivalent to A and V waves

A male infant was delivered via a caesarean section at 27 weeks of gestation, weighing 1283 g with 1- and 5-minute Apgar scores of 2 and 5, respectively. Umbilical artery pH was 7.197. He experienced intrauterine infection due to chorioamnionitis and showed prolonged prothrombin time international normalized ratio (PT-INR; 2.98) and moderate thrombocytopenia (14.0 × 10^3^/µl). Antibiotics and fresh frozen plasma were administered. Continuous flat flow (grade 0) of the ICV was confirmed at 10 hours after birth (Fig. 1a). At 18 hours after birth, his systemic blood pressure dropped to approximately 20 mmHg and dopamine was initiated. At 31 hours after birth, he showed a grade III IVH on the left side with partially interrupted ICV flow (grade 3; Fig. 1b). At 43 hours after birth, although PT-INR improved (1.84), he showed a grade III IVH on the contralateral side with PRF (Fig. 1c). PRF reappeared at 66 and 90 hours after birth (Fig. 1d). These PRFs were similar in shape to a triphasic venous flow pattern. On day 7, the ICV flow turned into a mild pulsatile pattern (grade 1) and the patient’s condition improved. The transition of the ICV flow pattern and IVH detection time are shown in Table 1a.

**Table 1:** The transition of the ICV flow pattern and time of detecting IVH in case 1

**(a) Case 1**							
**times after birth**	**10 hours**	**31 hours**	**43 hours**	**66 hours**	**90 hours**	**Day 7**	
ICV flow pattern	Grade 0 (Fig. 1a)	Grade 3 (Fig. 1b)	**PRF** (Fig. 1c)	**PRF**	**PRF** (Fig. 1d)	Grade 1	
IVH		Left Grade III	Right Grade III				
**(b) Case 2**							
**times after birth**	**5 hours**	**25 hours**	**41 hours**	**60 hours**	**87 hours**	**106 hours**	**Day 5**
ICV flow pattern	Grade 0 (Fig. 2a)	Grade 3	Grade 3 (Fig. 2b)	Grade 3	**PRF** (Fig. 2c)	Grade 2	Grade 1
IVH			Right Grade I		Bilateral Grade III		

Abbreviations: *ICV* internal cerebral vein, *IVH* intraventricular hemorrhage

ICV flow pattern: Grade 0, continuous flat flow; Grade 1, a mild pulsatile flow (minimum speed/maximum speed ≧ 0.5); Grade 2, severe pulsatile flow (minimum speed/maximum speed < 0.5); Grade 3, a partially interrupted flow (minimum speed = 0 cm/s); PRF, a partially reversed ICV blood flow

### Case 2

**Fig. 2:**
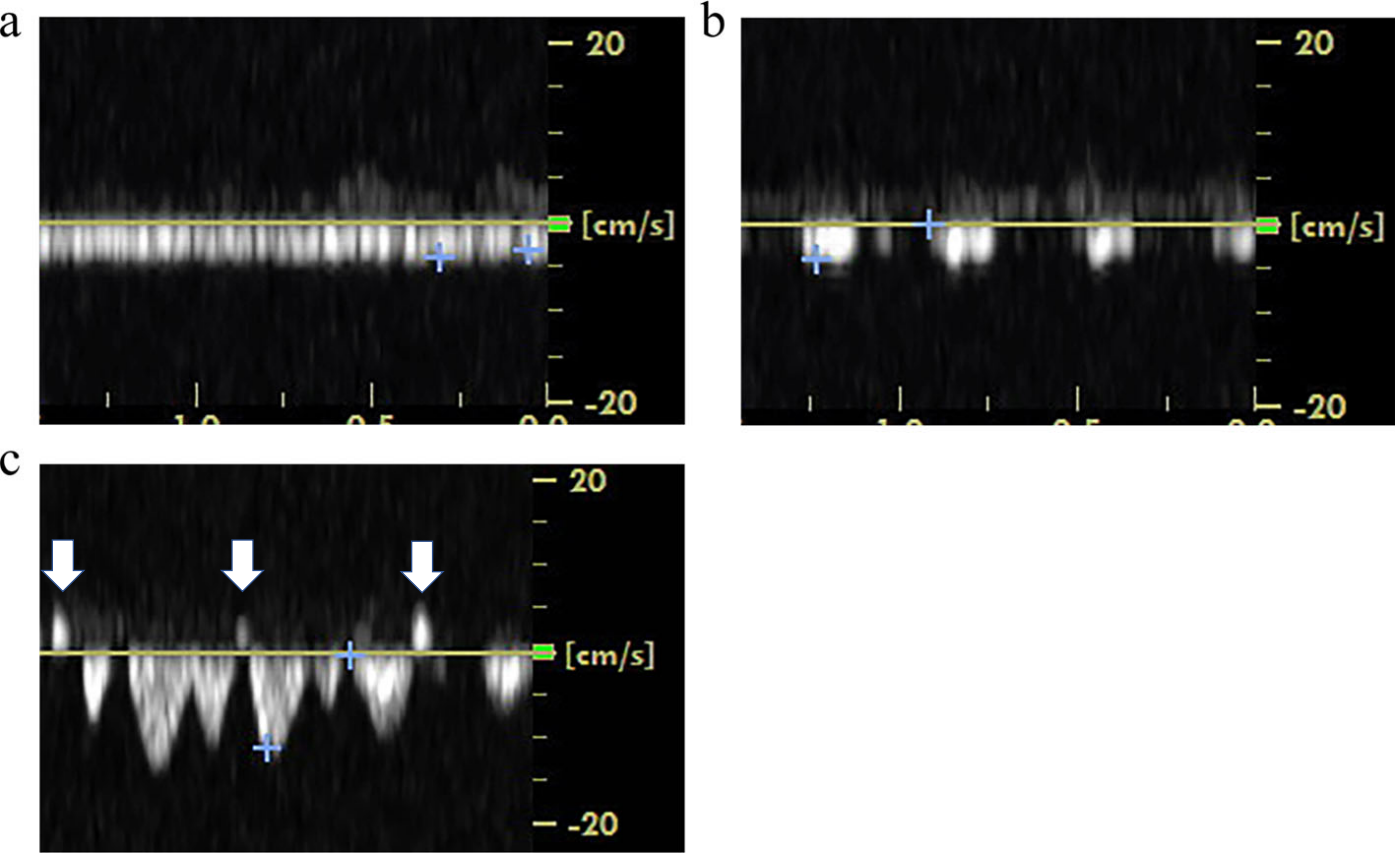
The flow of the internal cerebral vein in case 2. **a** At 5 hours after birth, the internal cerebral vein (ICV) flow pattern is continuous flat flow (grade 0). **b** Partially interrupted flow (grade 3) is confirmed at 41 hours after birth with grade I intraventricular hemorrhage (IVH) on the right side. **c** Partially reversed internal cerebral vein flow (PRF) (arrow) is recognized at 87 hours after birth with grade III bilateral IVH

A female infant was delivered via a caesarean section at 25 weeks gestation, weighing 760 g with 1- and 5-minute Apgar scores of 2 at both times. Umbilical artery pH was 7.221. Her PT-INR and platelet count were normal. She was provided with cardiac compression and artificial ventilation due to bradycardia immediately after birth. In the first ultrasound examination at 5 hours after birth, the ICV demonstrated a continuous flat flow (grade 0; Fig. 2a). A partially interrupted flow (grade 3) was confirmed at 25 hours after birth. At 41 hours after birth, she had a grade I IVH on the right side with partially interrupted ICV flow (grade 3; Fig. 2b). At 60 hours, a partially interrupted flow (grade 3) continued. At 87 hours, she had a bilateral grade III IVH with PRF of the ICV (Fig. 2c). At 106 hours, the ICV flow improved and changed to a severe pulsatile flow (grade 2). On day 5, the ICV flow turned into a mild pulsatile flow (grade 1). The transition of the ICV flow pattern and IVH detection time are shown in Table 1b.

## Discussion and conclusion

In both cases, the ICV flows were normal flat patterns initially and changed to partially interrupted flow or PRF at the same time as IVH detection, but were resolved after the development of IVH. This fact indicates that a change of the ICV flow might be related to IVH.

The PRF in these cases might have been derived from the pressure fluctuations of the right atrium. We determined that PRF had a triphasic waveform based on the pulsation of the arterial flow which was detected simultaneously in case (Fig. 1d) and was composed of four wave patterns: A-, S-, V-, and D-waves [7, 8]. The reverse flow corresponded approximately to the A- and V-waves, which are generated by elevation of the right atrial pressure [9]. It has been reported that fluctuations in central venous pressure may be transmitted intracranially since there is no valve in the jugular venous system [10]. An increase of venous pulsation is mostly caused by augmented atrial contraction waves which are derived from increased atrial pressure [11, 12]. Further deterioration causes a reversed A-wave [11, 13]. Namely, the pulsation and interruption of the ICV flow reported by Ikeda et al. [3] may have emerged during elevation of the right atrial pressure, which suggests the elevation of central venous pressure. Additionally, it is possible that PRF, which we newly report in this article, is the most severe pattern of ICV pulsation.

IVH is venous in origin [14] and may be caused by increased venous pressure. Ghazi-Birry et al. [5] reported that the venous vessels within the germinal matrix of hemorrhagic cases were invariably distorted, and the distortion of the venous vessels probably reflects the increase in central venous pressure. There are reports that IVH develops as a complication of sinovenous thrombosis and that the rupture of the small blood vessels which lead to IVH may be caused by increased venous pressure due to poor drainage of the vein of Galen or straight sinus [15]. As mentioned above, augmentation of venous pulsation indicates increased central venous pressure, and therefore, the pulsation of the ICV may be a sign of IVH. Especially, PRF, which is mainly caused by a reversed A-wave, may be a more severe sign [11].

It has been well established that cerebral vein vessels, vein sinuses, and blood flow velocities can be visualized using Doppler sonography [16], with successful visualization of all reported cases and velocity measured in > 80% of cases (Right side: 85.45%, Left side: 89.09%). Intracranial venous pulsations increase as the flow runs from the periphery towards the central portion [17]. For that reason, pulsation of the ICV may be augmented more in the proximal portion than the distal portion, and it is possible to overestimate ICV pulsation if the measurement site is near the great vein of Galen. Therefore, the measurement site of the ICV should be fixed.

This case report has some limitations. First, PRF was recognized concurrently with the detection of IVH in these cases. It is possible that subependymal bleeding may cause flow reversal by compressing the ICV. Ikeda et al. reported that severe pulsation or interruption of ICV flow was recognized before the detection of IVH in six out of eight cases and concurrently in one case [3]. Hence, PRF may be detectable with frequent examination before IVH development. Second, there were other risk factors of IVH in these cases such as birth asphyxia, coagulopathy, and thrombocytopenia in case 1 and severe birth asphyxia in case 2. However, disagreement exists concerning the role of coagulopathy in the causation of IVH, and the mechanism between IVH and thrombocytopenia has not yet been elucidated [2]. In these cases, birth asphyxia may have caused cardiac dysfunction that resulted in IVH due to increased central venous pressure [18]. Lastly, we screened the right and left ICVs in all cases and chose whichever side showed a sharper shape. However, the ICV of both sides merge into the great vein of Galen, so almost the same pressure may be applied to both sides. Thus, the ICV flow pattern may be an approximation unless there is a significant difference of vessel compliance or diameter between both sides [19]. Therefore, future studies are needed to investigate any simultaneous difference in ICV pulsation between the right and left sides.

For the strategy to prevent IVH, there is suggestive literature. There was a report of pulsatile superior sagittal sinus flow in an infant who had a large bilateral pleural effusion [10]. After the removal of effusions, the pulsation resolved. This suggests that decreasing the intrathoracic pressure could improve the pulsation of cerebral veins. Similarly, when severe pulsation, partial interruption, or PRF waves, which may indicate the elevation of central venous pressure, are recognized, load reduction therapy to decrease venous pressure might improve pulsation of the ICV and prevent IVH.

ICV flow patterns may be related to IVH in VLBW infants. PRF is a critical sign because it was found at the same time as the detection of IVH and improved after the development of IVH in these two cases. Our findings suggest that a change in ICV flow pattern may reflect the elevation of central venous pressure; thus, research on therapeutic strategies to decrease venous pressure in order to prevent IVH is needed.

## Data Availability

The datasets used and/or analyzed during the current study are available from the corresponding author on reasonable request.

## Abbreviations

IVH: intraventricular hemorrhage
VLBW: very low birth weight
ICV: internal cerebral vein
PRF: partially reversed internal cerebral vein flow
PT-INR: prothrombin time international normalized ratio
